# Publisher’s Note: We Changed Page Numbers to Article Numbers for Articles in *Neurology International* Volume 1–Volume 12, Issue 2

**DOI:** 10.3390/neurolint14010020

**Published:** 2022-03-01

**Authors:** Neurology International Editorial Office

**Affiliations:** MDPI AG, St. Alban-Anlage 66, 4052 Basel, Switzerland; neurolint@mdpi.com

*Neurology International* [1] Volumes 1 (2009) to 12, Issue 2 (2020) were published by PAGEPress. *Neurology International* Volume 12, Issue 3 (2020) onwards is being published by MDPI.

When it comes to Volume 1–Volume 12, Issue 2, those articles that were originally published by PAGEPress in Open Access under a CC-BY (or CC-BY-NC-ND) license, will now be hosted by MDPI on mdpi.com, as a courtesy and upon agreement with PAGEPress.

To standardize the metadata format of all previous articles, MDPI has republished 204 articles in Volume 1–Volume 12, Issue 2 with article numbers replacing page numbers (Table 1).

## Figures and Tables

**Table 1:** MDPI changed page numbers to articles numbers for 238 articles in Volume 1–Volume 12, Issue 2.

DOI	Previous Page Number	Current Article Number
10.4081/ni.2009.e1	1–3	e1
10.4081/ni.2009.e2	4–6	e2
10.4081/ni.2009.e3	7–9	e3
10.4081/ni.2009.e4	10–14	e4
10.4081/ni.2009.e5	15–18	e5
10.4081/ni.2009.e6	19–23	e6
10.4081/ni.2009.e7	24–26	e7
10.4081/ni.2009.e8	27–31	e8
10.4081/ni.2009.e9	32–35	e9
10.4081/ni.2009.e10	36–40	e10
10.4081/ni.2009.e11	41–43	e11
10.4081/ni.2009.e12	44–45	e12
10.4081/ni.2009.e13	46–51	e13
10.4081/ni.2009.e14	52–53	e14
10.4081/ni.2009.e15	54–56	e15
10.4081/ni.2009.e16	57–60	e16
10.4081/ni.2009.e17	61–62	e17
10.4081/ni.2009.e18	63–64	e18
10.4081/ni.2009.e19	65–71	e19
10.4081/ni.2009.e20	72–78	e20
10.4081/ni.2009.e22	82–83	e22
10.4081/ni.2010.e1	1–6	e1
10.4081/ni.2010.e2	7–13	e2
10.4081/ni.2010.e3	14–19	e3
10.4081/ni.2010.e4	20–23	e4
10.4081/ni.2010.e5	24–27	e5
10.4081/ni.2010.e6	28–32	e6
10.4081/ni.2010.e7	33–35	e7
10.4081/ni.2010.e8	36–37	e8
10.4081/ni.2010.e9	38–41	e9
10.4081/ni.2010.e10	42–47	e10
10.4081/ni.2010.e11	48–51	e11
10.4081/ni.2010.e13	57–61	e13
10.4081/ni.2010.e14	62–64	e14
10.4081/ni.2010.e15	65	e15
10.4081/ni.2010.e16	66–68	e16
10.4081/ni.2010.e17	69–71	e17
10.4081/ni.2010.e18	73–76	e18
10.4081/ni.2010.e19	77	e19
10.4081/ni.2011.e1	1–3	e1
10.4081/ni.2011.e2	4–8	e2
10.4081/ni.2011.e3	9–11	e3
10.4081/ni.2011.e4	12–16	e4
10.4081/ni.2011.e5	17–21	e5
10.4081/ni.2011.e6	22–23	e6
10.4081/ni.2011.e7	24–27	e7
10.4081/ni.2011.e8	28–30	e8
10.4081/ni.2011.e9	31–34	e9
10.4081/ni.2011.e10	35–39	e10
10.4081/ni.2011.e11	41–44	e11
10.4081/ni.2011.e12	45–49	e12
10.4081/ni.2011.e13	50–56	e13
10.4081/ni.2011.e14	57–59	e14
10.4081/ni.2011.e15	60–67	e15
10.4081/ni.2011.e16	68–69	e16
10.4081/ni.2012.e1	1–3	e1
10.4081/ni.2012.e2	4–8	e2
10.4081/ni.2012.e3	9–14	e3
10.4081/ni.2012.e4	15–19	e4
10.4081/ni.2012.e5	20–23	e5
10.4081/ni.2012.e6	24–25	e6
10.4081/ni.2012.e7	26–31	e7
10.4081/ni.2012.e8	32–34	e8
10.4081/ni.2012.e9	35–39	e9
10.4081/ni.2012.e10	40–43	e10
10.4081/ni.2012.e11	44–48	e11
10.4081/ni.2012.e12	49–51	e12
10.4081/ni.2012.e13	52–59	e13
10.4081/ni.2012.e14	60–64	e14
10.4081/ni.2012.e15	65–70	e15
10.4081/ni.2012.e16	71–77	e16
10.4081/ni.2012.e17	78–84	e17
10.4081/ni.2012.e18	85–86	e18
10.4081/ni.2012.e19	87–89	e19
10.4081/ni.2013.e1	1–5	e1
10.4081/ni.2013.e2	6–7	e2
10.4081/ni.2013.e3	8–9	e3
10.4081/ni.2013.e4	10–12	e4
10.4081/ni.2013.e5	13–16	e5
10.4081/ni.2013.e6	17–19	e6
10.4081/ni.2013.e7	20–22	e7
10.4081/ni.2013.e8	23–27	e8
10.4081/ni.2013.e9	28–30	e9
10.4081/ni.2013.e10	31–33	e10
10.4081/ni.2013.e11	34–36	e11
10.4081/ni.2013.e12	37–40	e12
10.4081/ni.2013.e13	41–44	e13
10.4081/ni.2013.e14	45–47	e14
10.4081/ni.2013.e15	48–52	e15
10.4081/ni.2013.e16	53–57	e16
10.4081/ni.2013.e17	58–61	e17
10.4081/ni.2013.e18	62–64	e18
10.4081/ni.2013.e19	65–68	e19
10.4081/ni.2013.e20	69–72	e20
10.4081/ni.2013.e21	73–74	e21
10.4081/ni.2013.e22	75–78	e22
10.4081/ni.2013.e23	79–83	e23
10.4081/ni.2013.e24	84–88	e24
10.4081/ni.2014.5048	1–3	5048
10.4081/ni.2014.5133	70–73	5133
10.4081/ni.2014.5140	6–7	5140
10.4081/ni.2014.5208	47–51	5208
10.4081/ni.2014.5307	4–5	5307
10.4081/ni.2014.5347	25–26	5347
10.4081/ni.2014.5348	29–31	5348
10.4081/ni.2014.5349	27–28	5349
10.4081/ni.2014.5352	23–24	5352
10.4081/ni.2014.5359	12–14	5359
10.4081/ni.2014.5367	36–39	5367
10.4081/ni.2014.5369	19–22	5369
10.4081/ni.2014.5376	11–13	5376
10.4081/ni.2014.5385	15–18	5385
10.4081/ni.2014.5388	32–35	5388
10.4081/ni.2014.5395	40–42	5395
10.4081/ni.2014.5442	66–69	5442
10.4081/ni.2014.5487	53–55	5487
10.4081/ni.2014.5492	43–46	5492
10.4081/ni.2014.5519	52	5519
10.4081/ni.2014.5521	61–65	5521
10.4081/ni.2014.5547	56–60	5547
10.4081/ni.2014.5553	74–77	5553
10.4081/ni.2014.5620	83–85	5620
10.4081/ni.2014.5662	78–80	5662
10.4081/ni.2014.5716	81–82	5716
10.4081/ni.2015.5417	28–30	5417
10.4081/ni.2015.5430	2–7	5430
10.4081/ni.2015.5452	1	5452
10.4081/ni.2015.5770	22–24	5770
10.4081/ni.2015.5807	36–38	5807
10.4081/ni.2015.5809	8–11	5809
10.4081/ni.2015.5840	19–21	5840
10.4081/ni.2015.5885	39–47	5885
10.4081/ni.2015.5886	31–35	5886
10.4081/ni.2015.5952	48–53	5952
10.4081/ni.2015.5962	58–61	5962
10.4081/ni.2015.5966	25–27	5966
10.4081/ni.2015.5971	12–14	5971
10.4081/ni.2015.5973	15–18	5973
10.4081/ni.2015.6057	62–65	6057
10.4081/ni.2015.6079	54–57	6079
10.4081/ni.2015.6133	66–69	6133
10.4081/ni.2015.6207	70–73	6207
10.4081/ni.2016.5846	80–83	5846
10.4081/ni.2016.5939	14–22	5939
10.4081/ni.2016.6104	9–11	6104
10.4081/ni.2016.6132	57–59	6132
10.4081/ni.2016.6310	23–26	6310
10.4081/ni.2016.6311	7–8	6311
10.4081/ni.2016.6322	36–38	6322
10.4081/ni.2016.6330	69–73	6330
10.4081/ni.2016.6361	67–68	6361
10.4081/ni.2016.6365	4–6	6365
10.4081/ni.2016.6416	42–48	6416
10.4081/ni.2016.6428	12–13	6428
10.4081/ni.2016.6444	1–3	6444
10.4081/ni.2016.6513	39–41	6513
10.4081/ni.2016.6534	33–35	6534
10.4081/ni.2016.6553	74–76	6553
10.4081/ni.2016.6617	27–29	6617
10.4081/ni.2016.6661	60–63	6661
10.4081/ni.2016.6676	64–66	6676
10.4081/ni.2016.6677	54–56	6677
10.4081/ni.2016.6812	49–53	6812
10.4081/ni.2016.6822	77–79	6822
10.4081/ni.2016.6841	84–87	6841
10.4081/ni.2017.6904	17–19	6904
10.4081/ni.2017.6920	11–16	6920
10.4081/ni.2017.6933	1–7	6933
10.4081/ni.2017.6957	8–10	6957
10.4081/ni.2017.7015	25–28	7015
10.4081/ni.2017.7032	21–24	7032
10.4081/ni.2017.7043	29–33	7043
10.4081/ni.2017.7133	34–35	7133
10.4081/ni.2017.7216	63–68	7216
10.4081/ni.2017.7276	38–40	7276
10.4081/ni.2017.7279	79–81	7279
10.4081/ni.2017.7280	69–70	7280
10.4081/ni.2017.7312	36–37	7312
10.4081/ni.2017.7336	71–74	7336
10.4081/ni.2017.7343	75–78	7343
10.4081/ni.2017.7356	82–83	7356
10.4081/ni.2018.6993	13–17	6993
10.4081/ni.2018.7326	111–117	7326
10.4081/ni.2018.7385	20–23	7385
10.4081/ni.2018.7436	65–68	7436
10.4081/ni.2018.7468	1–2	7468
10.4081/ni.2018.7469	57–60	7469
10.4081/ni.2018.7473	3–12	7473
10.4081/ni.2018.7510	24–27	7510
10.4081/ni.2018.7516	32–34	7516
10.4081/ni.2018.7536	45–50	7536
10.4081/ni.2018.7558	35–40	7558
10.4081/ni.2018.7562	18–19	7562
10.4081/ni.2018.7563	97–98	7563
10.4081/ni.2018.7599	41–44	7599
10.4081/ni.2018.7625	28–31	7625
10.4081/ni.2018.7638	61–64	7638
10.4081/ni.2018.7665	51–53	7665
10.4081/ni.2018.7666	90–92	7666
10.4081/ni.2018.7690	79–82	7690
10.4081/ni.2018.7694	54–56	7694
10.4081/ni.2018.7729	69–73	7729
10.4081/ni.2018.7737	77–78	7737
10.4081/ni.2018.7761	83–89	7761
10.4081/ni.2018.7780	74–76	7780
10.4081/ni.2018.7852	95–96	7852
10.4081/ni.2018.7867	93–94	7867
10.4081/ni.2018.7877	103–106	7877
10.4081/ni.2018.7917	99–102	7917
10.4081/ni.2018.7921	107–110	7921
10.4081/ni.2019.7815	4–8	7815
10.4081/ni.2019.7941	12–14	7941
10.4081/ni.2019.7958	1–3	7958
10.4081/ni.2019.7959	9–11	7959
10.4081/ni.2019.7983	24–29	7983
10.4081/ni.2019.8001	15–17	8001
10.4081/ni.2019.8079	18–20	8079
10.4081/ni.2019.8102	21–23	8102
10.4081/ni.2019.8129	33–36	8129
10.4081/ni.2019.8177	66–69	8177
10.4081/ni.2019.8180	30–32	8180
10.4081/ni.2019.8182	40–43	8182
10.4081/ni.2019.8191	38–39	8191
10.4081/ni.2019.8198	52–57	8198
10.4081/ni.2019.8205	75–77	8205
10.4081/ni.2019.8207	58–62	8207
10.4081/ni.2019.8241	47–49	8241
10.4081/ni.2019.8253	44–46	8253
10.4081/ni.2019.8257	50–51	8257
10.4081/ni.2019.8282	70–74	8282
10.4081/ni.2019.8318	63–65	8318
10.4081/ni.2020.8292	1–5	8292
10.4081/ni.2020.8328	6–8	8328
10.4081/ni.2020.8346	19–23	8346
10.4081/ni.2020.8401	9–14	8401
10.4081/ni.2020.8558	29–32	8558
10.4081/ni.2020.8639	15–18	8639
10.4081/ni.2020.8652	24–28	8652
